# Retrospective analysis of micronutrient status and prostate health: linking serum zinc, selenium, and vitamin D levels to prostate-specific antigen and urinary tract function

**DOI:** 10.3389/fmolb.2026.1779179

**Published:** 2026-08-21

**Authors:** Changfu Feng, Xiaojing Liu, Subi Nijiati, Xiangbo Ma, Honglin Yang

**Affiliations:** Urumqi Friendship Hospital, Xinjiang, China

**Keywords:** lower urinary tract symptoms, prostate health, prostate-specific antigen, retrospective study, selenium, vitamin D, zinc

## Abstract

**Background:**

Micronutrients such as zinc, selenium, and vitamin D play a crucial role in prostate physiology. Deficiencies in these nutrients may be associated with prostate-specific antigen (PSA) levels and with impaired urinary tract parameters, including the International Prostate Symptom Score (IPSS), maximum urinary flow rate (Qmax), and post-void residual (PVR) volume. This retrospective study evaluated the association between serum micronutrient status and prostate health indicators in adult men.

**Methods:**

Medical records of 400 men aged ≥45 years who underwent prostate evaluation between 2019 and 2022 were retrospectively analyzed. Serum zinc, selenium, and 25-hydroxyvitamin D levels were recorded alongside PSA values and urinary tract parameters, including IPSS, Qmax, and PVR volume. Based on reference ranges, participants were categorized into low- or normal-micronutrient groups. Correlation analyses and multivariable logistic regression were performed to identify independent predictors of elevated PSA (>4 ng/mL) and severe urinary symptoms (IPSS ≥20).

**Results:**

120 participants exhibited low micronutrient levels. Compared with men with normal levels, this group showed significantly higher PSA (5.2 ± 2.8 vs. 2.9 ± 1.5 ng/mL), higher IPSS scores (16.5 ± 5.8 vs. 10.2 ± 5.2), lower Qmax (11.2 ± 4.1 vs. 16.1 ± 4.7 mL/s), and higher PVR (61.7 ± 20.3 vs. 42.2 ± 18.5 mL) (all p < 0.001). Serum zinc, selenium, and vitamin D levels were inversely correlated with PSA and IPSS and positively correlated with Qmax (all p < 0.001). In multivariable regression analysis, low zinc (OR 2.48; 95% CI: 1.45–4.23), low selenium (OR 2.12; 95% CI: 1.27–3.52), and low vitamin D (OR 1.87; 95% CI: 1.11–3.16) were independent predictors of elevated PSA.

**Conclusion:**

Micronutrient deficiencies were associated with altered PSA levels and impaired urinary tract function. Monitoring and correcting micronutrient deficiencies may represent a valuable component of prostate health management, though prospective studies are needed to confirm causality.

## Graphical Abstract

**Figure:**
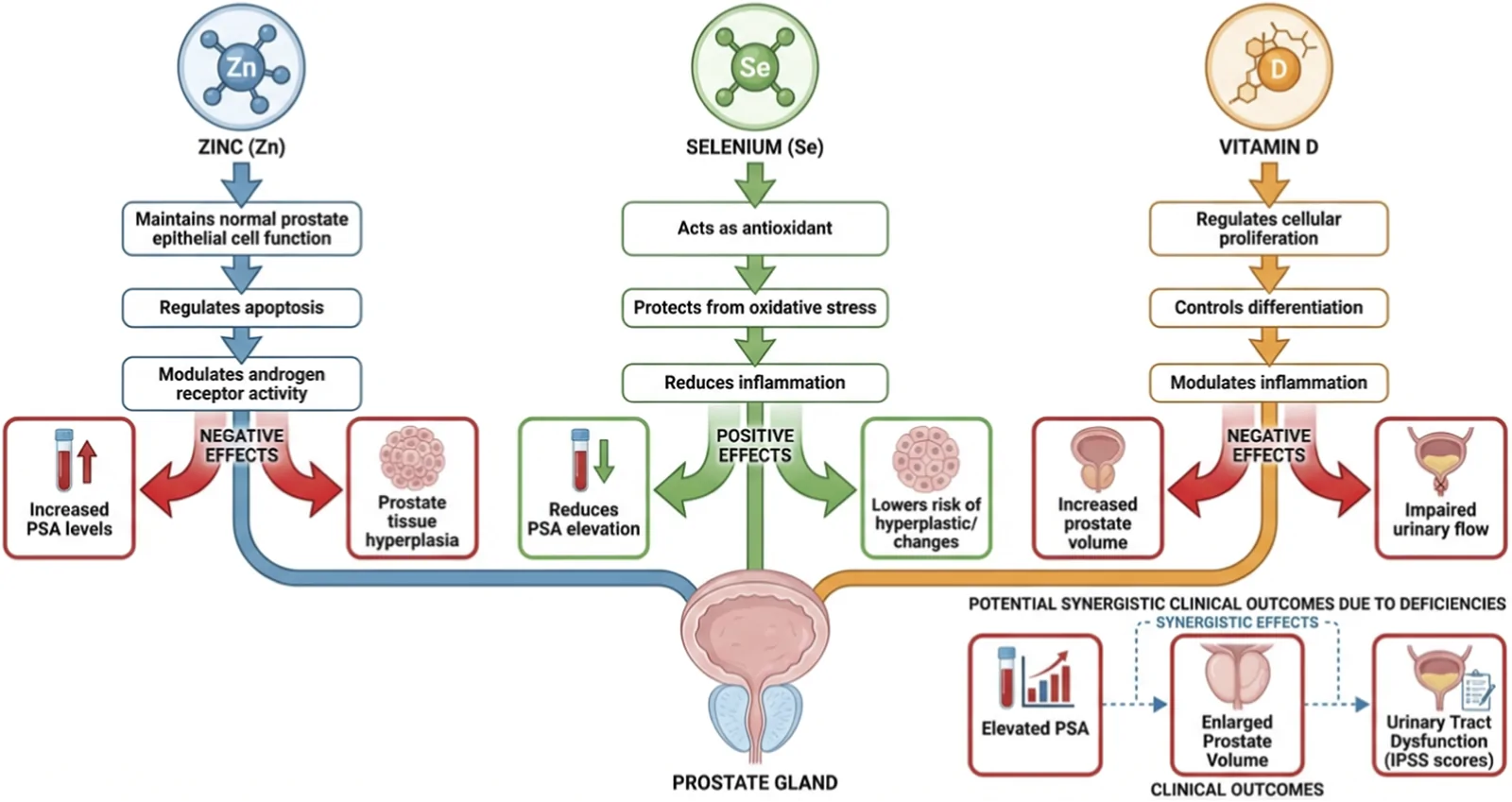

## 1 Introduction

Some of the most common urological diseases affecting older men are prostate illnesses such as benign prostatic hyperplasia (BPH), elevated levels of PSA (a marker of prostate activity), and lower urinary tract symptoms (LUTS) (Tang et al., 2023). After the fifth decade of life, the prevalence gradually increases worldwide due to age-related hormonal changes, persistent low-grade inflammation, and gradual shifts in prostate tissue structure (Vuichoud and Loughlin, 2015). The necessity to comprehend biological aspects that may affect prostate function is highlighted by the fact that these diseases may frequently contribute to urinary blockage, decreased urine flow, and symptom burden. Micronutrient status has recently attracted interest as a potential modulator of urological health. Zinc, selenium, and vitamin D have attracted particular attention among nutrients for their well-established physiological roles in immunological regulation, antioxidant defense, and epithelial maintenance (Hu et al., 2024). Zinc is highly concentrated in the healthy prostate and is essential for citrate metabolism, mitochondrial energy production, and epithelial cell integrity. Zinc status may be related to prostate physiology, according to several clinical and biochemical investigations that have found changed zinc values in males with prostate hypertrophy or dysfunction (Zaichick, 2021).

Selenium is also a necessary trace element that helps defend against oxidative stress and regulate inflammatory reactions. Decreased concentrations of selenium have been linked to indicators of prostate stress or dysfunction, according to studies conducted in the last 10 years, albeit results vary by population (Jiang et al., 2023). Selenium remains under study for its potential role in preserving cellular redox homeostasis in prostatic tissue, despite conflicting findings. The relationship between vitamin D and prostate health has been further investigated (Kang et al., 2024). Prostate cells express vitamin D receptors, and research suggests these receptors may influence immune responses, smooth muscle function, and cell growth regulation. Reduced quantities of vitamin D have been linked in clinical trials to higher PSA readings, larger prostate volumes, or more severe symptoms, indicating potential significance in early prostate alterations (Reddy et al., 2024).

There is insufficient scientific literature assessing zinc, selenium, and vitamin D collectively in the same population, despite each micronutrient having been investigated separately. Additionally, only a small number of studies have linked these micronutrient profiles to a wide range of prostate health metrics, including objective measures of urinary tract function, biochemical markers, and symptom scores (Na et al., 2026; Hou et al., 2026). This disparity emphasizes the need for more comprehensive evaluations that examine the relationships between various micronutrient deficiencies and clinical indicators of lower urinary tract and prostate health (Yang J. et al., 2025; Guo et al., 2025; Yang Z. et al., 2025). In addition, although zinc, selenium, and vitamin D have been investigated separately, their combined effects may be synergistic, particularly in regulating oxidative stress, immune function, and the prostate epithelium. Moreover, selenium exhibits a U-shaped dose–response curve, with potentially adverse effects at both low and high concentrations, suggesting a need for a balanced approach in clinical research. To close this gap, the current retrospective study examines the relationships between serum zinc, selenium, and vitamin D levels and PSA, urinary symptom load, and urinary function indicators in adult males. The investigation aims to provide a broader view of micronutrient status in the context of prostate health by examining multiple micronutrients simultaneously.

## 2 Methodology

### 2.1 Study design

A retrospective, observational study was conducted using medical records from a tertiary-level urology center. The analysis incorporated males who underwent regular prostate examination between January 2019 and December 2022. The Ethics Committee of Friendship Hospital approved this research (approval number: FH2025024).

### 2.2 Study population

This research included male subjects aged 45 years and older with accessible serum zinc, selenium, and vitamin D levels, PSA testing, and an assessment of lower urinary tract symptoms. Patients with previous instances of prostate cancer, acute or chronic prostatitis, urinary tract infections, prior prostate surgery, or chronic kidney disease stage 3 or higher were omitted. Additionally, those with incomplete laboratory or urological records and those who had consumed micronutrient supplements in the previous 6 months were excluded.

### 2.3 Assessment of serum micronutrient status

Micronutrient assessment was based on laboratory data from standard clinical treatment. The hospital laboratory used standardized protocols to evaluate fasting venous blood samples. Atomic absorption spectrophotometry was used to quantify zinc and selenium in serum. In contrast, a chemiluminescent immunoassay was used to quantify 25-hydroxyvitamin D levels. Zinc: 80–120 μg/dL; selenium: 90–130 μg/L; and vitamin D: 20–50 ng/mL were used as reference ranges. Individuals were categorized as having low micronutrient status if one or more micronutrient values were below the lower reference range. The composite classification was further broken down into individual micronutrients (zinc, selenium, and vitamin D). A cumulative deficiency score (0–3) was also created based on the number of deficient micronutrients to assess dose–response effects. In addition to the composite classification, individual micronutrient deficiencies (zinc, selenium, and vitamin D) were also analyzed separately to evaluate their independent associations with prostate outcomes. The composite low-micronutrient variable was initially created to evaluate the cumulative effect of micronutrient insufficiency and to improve statistical power in assessing the overall burden of nutritional deficiency.

### 2.4 Prostate and urinary tract outcome measures

An electrochemiluminescence immunoassay was used to measure PSA levels, and results were obtained from the medical laboratory. PSA levels were deemed elevated if greater than 4 ng/mL and very elevated if greater than 10 ng/mL. The IPSS, obtained during the clinical assessment, was used to evaluate lower urinary tract symptoms. Qmax, determined by uroflowmetry, and PVR, determined by bladder ultrasonography, were two objective urinary measures. An IPSS score ≥20 was considered severe urine symptoms, while a PVR score>100 mL was considered clinically substantial urinary retention.

### 2.5 Covariates

The health records were used to gather data on age, BMI, smoking habits, type 2 diabetes, high blood pressure, and any family history of prostate illness. These variables were included as potential confounders in the multivariable models and in the descriptive analyses. Some clinically important variables, including prostate volume, medications, inflammatory markers, renal function, and detailed lifestyle factors, were not always available and were therefore excluded.

### 2.6 Statistical analysis

Data are presented as mean ± standard deviation or as frequencies and percentages. The Shapiro-Wilk test and histograms were used to check for normality. Independent-samples t-tests, chi-square tests, and Pearson correlations were performed where appropriate. Due to the possibility of skewed distributions in PSA and post-void residual (PVR), these variables were examined. Binary logistic regression models were used to assess variables independently associated with elevated PSA (>4 ng/mL) and severe LUTS (IPSS ≥20), controlling for potential confounders such as age, body mass index (BMI), diabetes, hypertension, smoking, and family history of prostate cancer. Low micronutrient status was examined as a binary variable (at least one low micronutrient) and as individual micronutrient levels. The variance inflation factor (VIF) was used to assess multicollinearity, and a VIF <5 was considered acceptable. All results are reported as odds ratios (ORs) and 95% confidence intervals (CIs). A complete case analysis was performed to address missing data. A p-value of <0.05 was considered statistically significant.

## 3 Results

### 3.1 Baseline demographic characteristics

There were 400 participants in the study, with 120 in the Low Micronutrient Group and 280 in the Normal Micronutrient Group. Overall, there were many differences in demographics and clinical data between the two groups. The average age of all participants was 62.5 ± 8.7 years, whereas those with low micronutrient levels were significantly older (64.2 ± 8.9 years) than those with normal micronutrient levels (61.5 ± 8.5 years; p = 0.02). The two groups also differed in body mass index, with a slightly higher mean BMI in the low-micronutrient group (27.8 ± 3.6 kg/m^2^) compared with the normal group (26.9 ± 3.3 kg/m^2^); the difference was borderline statistically significant (p = 0.05). The prevalence of diabetes was more than double in the group with low micronutrient status (25%) than in the group with normal levels (17.1%; p = 0.04). Although the differences were not significant, hypertension (33.3% versus 25%; p = 0.08) and smoking (29.2% versus 21.4%; p = 0.07) were more prevalent in the low micronutrient group. These points point towards possible lifestyle and metabolic influences. Moreover, the prevalence of family history of prostate disease was significantly higher in the deficient group (15%) than in the normal one (8.6%; p = 0.03). In general, the low-micronutrient group consisted of older individuals with more comorbidities, suggesting a greater need to control for these variables in future analyses (Table 1).

**TABLE 1:** Baseline demographic characteristics.

Characteristic	Total (n = 400)	Low micronutrient group (n = 120)	Normal micronutrient group (n = 280)	p-value
Age (years, mean ± SD)	62.5 ± 8.7	64.2 ± 8.9	61.5 ± 8.5	0.02
BMI (kg/m^2^, mean ± SD)	27.2 ± 3.4	27.8 ± 3.6	26.9 ± 3.3	0.05
Diabetes, n (%)	78 (19.5%)	30 (25%)	48 (17.1%)	0.04
Hypertension, n (%)	110 (27.5%)	40 (33.3%)	70 (25%)	0.08
Smoking, n (%)	95 (23.8%)	35 (29.2%)	60 (21.4%)	0.07
Family history of prostate disease, n (%)	42 (10.5%)	18 (15%)	24 (8.6%)	0.03

### 3.2 Serum micronutrient levels

The mean serum zinc level in the overall cohort was 92.4 ± 18.7 μg/dL, selenium was 110.3 ± 22.4 μg/L, and vitamin D was 28.2 ± 9.5 ng/mL. According to the reference ranges, 30% (n = 120) of individuals had low micronutrient status, and 70% (n = 280) had normal micronutrient status. Within the population, we observed a distribution of micronutrient levels, with some participants’ levels below the clinical cut-offs for zinc, selenium, and vitamin D (Table 2).

**TABLE 2:** Serum micronutrient levels.

Micronutrient	Total (n = 400)	Low micronutrient group	Normal micronutrient group	Reference range	p-value
Zinc (μg/dL)	92.4 ± 18.7	74.5 ± 7.8	100.2 ± 12.1	80–120	<0.001
Selenium (μg/L)	110.3 ± 22.4	85.6 ± 9.7	120.1 ± 18.3	90–130	<0.001
Vitamin D (ng/mL)	28.2 ± 9.5	18.4 ± 3.7	32.1 ± 8.4	20–50	<0.001

### 3.3 Prostate-specific antigen (PSA) levels

Prostate-specific antigen (PSA) tests showed that the low- and normal-micronutrient groups differed significantly among cases, indicating that micronutrient levels might influence prostate condition, that is, a biomarker. The average PSA level in the whole group was 3.6 ± 2.1 ng/mL; nevertheless, those in the Low Micronutrient Group showed remarkably increased levels of PSA (5.2 ± 2.8 ng/mL) over those in the Normal Micronutrient Group (2.9 ± 1.5 ng/mL), with this difference being very statistically significant (p < 0.001). Clinically relevant PSA thresholds (>4 and >10 ng/mL) were used to categorize PSA as elevated and high risk, respectively. About one-fourth of all persons (23.8%) had PSA concentrations greater than 4 ng/mL, but the prevalence was much higher among participants with insufficient micronutrient intake (41.7%) than among those with adequate micronutrient intake (16.1%; p < 0.001). This puts forth the idea that deficiency of zinc, selenium, and vitamin D may be a factor in people’s early markers of prostate enlargement or even cancer. According to the even more critical cutoff of PSA >10 ng/mL, 7% of the entire sample could be classified as significantly higher risk patients. Furthermore, the percentage in the low-micronutrient group (12.5%) was significantly higher than that in the normal-micronutrient group (4.6%; p = 0.004) (Table 3).

**TABLE 3:** Prostate-specific antigen (PSA) levels.

Parameter	Total (n = 400) mean ± SD	Low micronutrient group mean ± SD	Normal micronutrient group mean ± SD	p-value	Median (IQR) total	Mann–Whitney U test p-value
PSA (ng/mL)	3.6 ± 2.1	5.2 ± 2.8	2.9 ± 1.5	<0.001	3.1 (2.0–4.8)	<0.001
PSA >4 ng/mL, n (%)	95 (23.8%)	50 (41.7%)	45 (16.1%)	<0.001	—	—
PSA >10 ng/mL, n (%)	28 (7.0%)	15 (12.5%)	13 (4.6%)	0.004	—	—

PSA, prostate-specific antigen.

### 3.4 Urinary tract function (IPSS, Qmax, PVR)

The evaluation of urinary tract function showed a significant deterioration in the Low Micronutrient Group compared to the Normal Micronutrient Group. The average International Prostate Symptom Score (IPSS). Nonetheless, participants with low micronutrient status had considerably greater symptom severity, with a mean IPSS score of 16.5 ± 5.8, compared with 10.2 ± 5.2 in the normal micronutrient group (p < 0.001). The objective assessment of urinary flow and bladder emptying also revealed great differences. The average maximum urine flow rate (Qmax) was significantly lower in the micronutrient-deficient groups (11.2 ± 4.1 mL/s) than in the normal group (16.1 ± 4.7 mL/s; p < 0.001), suggesting impaired urinary flow in people with micronutrient deficiencies. Residual urine volume after urination (PVR) also showed a similar pattern: the low micronutrient group had much higher PVR values (61.7 ± 20.3 mL) in contrast to the normal group (42.2 ± 18.5 mL; p < 0.001). PVR >100 mL, a sign of clinically significant urinary retention, was observed in 8% of the total population, but its occurrence was three times higher in the low-micronutrient group (15%) than in the normal group (5%; p = 0.002) (Tables 4, 5).

**TABLE 4:** Urinary tract function (IPSS, Qmax, PVR).

Parameter	Total (n = 400) mean ± SD	Low micronutrient group mean ± SD	Normal micronutrient group mean ± SD	p-value	Median (IQR) total	Mann–Whitney U test p-value
IPSS score	12.3 ± 6.5	16.5 ± 5.8	10.2 ± 5.2	<0.001	12 (8–17)	<0.001
Qmax (mL/s)	14.6 ± 5.2	11.2 ± 4.1	16.1 ± 4.7	<0.001	14.0 (11.0–18.0)	<0.001
PVR (mL)	48.3 ± 22.4	61.7 ± 20.3	42.2 ± 18.5	<0.001	45 (32–62)	<0.001
Urinary retention (PVR >100 mL), n (%)	32 (8.0%)	18 (15.0%)	14 (5.0%)	0.002	—	—

IPSS, international prostate symptom score, Qmax, maximum urinary flow rate, PVR, post-void residual.

**TABLE 5:** Spearman correlation analysis.

Variable	Spearman’s rho (ρ)	p-value
PSA vs. vitamin D	−0.41	<0.001
PSA vs. selenium	−0.35	<0.001
PSA vs. zinc	−0.38	<0.001
IPSS vs. vitamin D	−0.44	<0.001
Qmax vs. vitamin D	0.39	<0.001
PVR vs. vitamin D	−0.36	<0.001

### 3.5 Association between micronutrients and prostate outcomes

Correlation analysis revealed strong, significant associations between patients’ serum micronutrient levels and major prostate-related clinical outcomes, with zinc, selenium, and vitamin D showing significant associations via their physiological effects, thereby supporting prostate and urinary health through the consumption of these micronutrients. In continuous form, micronutrient concentrations showed similar associations with PSA and urinary measures, further enhancing confidence in the results. Among the micronutrients, zinc showed the strongest associations. In the case of zinc, higher concentrations are associated with decreased PSA levels (r = −0.42, p < 0.001) and a moderate decline in IPSS-related urinary symptoms (r = −0.35, p < 0.001). At the same time, there was a positive association between zinc levels and maximum urinary flow rate (Qmax) (r = 0.31, p < 0.001) and a negative correlation with post-void residual volume (PVR) (r = −0.29, p < 0.001). Thus, it has been implied that a sufficient zinc concentration could reduce urinary obstruction and, hence, improve urinary function, while not causing bladder outflow obstruction. Selenium levels showed a similar, albeit slightly weaker, pattern of associations. Higher selenium concentrations were significantly associated with lower PSA (r = −0.37, p < 0.001) and lower IPSS scores (r = −0.32, p < 0.001). Furthermore, Qmax (r = 0.28, p < 0.001) and PVR (r = −0.25, p < 0.001) were positively and negatively correlated with selenium, respectively, suggesting a beneficial role of selenium in urinary function, likely related to its antioxidant properties. Vitamin D levels were also inversely associated with PSA and symptom severity (r = −0.33, p < 0.001) and IPSS (r = −0.30, p < 0.001) while at the same time having positive associations with Qmax (r = 0.26, p < 0.001) and negative correlations with PVR (r = −0.23, p < 0.001). (Table 6).

**TABLE 6:** Correlation between micronutrients and prostate outcomes.

Micronutrient	PSA	IPSS	Qmax	PVR
Zinc (μg/dL)	r = −0.42, p < 0.001	r = −0.35, p < 0.001	r = 0.31, p < 0.001	r = −0.29, p < 0.001
Selenium (μg/L)	r = −0.37, p < 0.001	r = −0.32, p < 0.001	r = 0.28, p < 0.001	r = −0.25, p < 0.001
Vitamin D (ng/mL)	r = −0.33, p < 0.001	r = −0.30, p < 0.001	r = 0.26, p < 0.001	r = −0.23, p < 0.001

### 3.6 Association between micronutrient deficiency and elevated PSA (>4 ng/mL)

**FIGURE 1:**
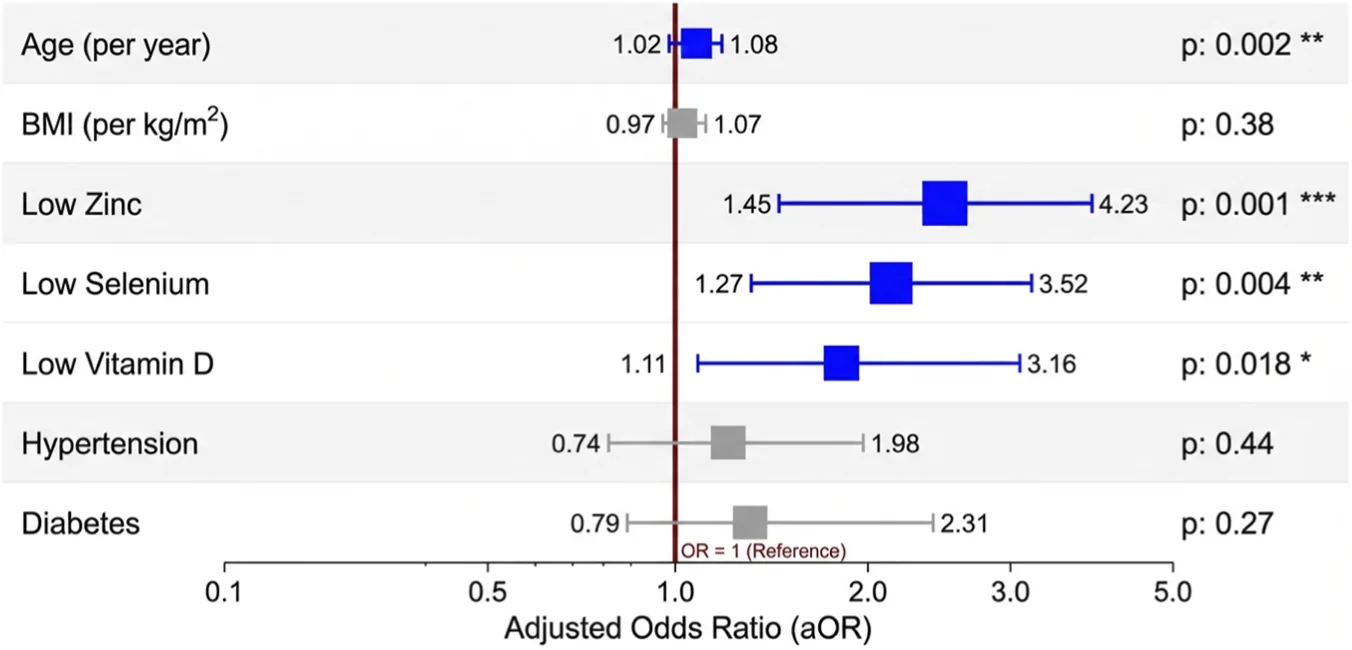
Multivariable regression analysis for elevated PSA (>4 ng/mL).

Multivariable logistic regression analysis was performed to identify independent predictors of elevated PSA (>4 ng/mL). The multivariable logistic regression analysis identified factors independently associated with the association between micronutrient deficiency and Elevated PSA (>4 ng/mL), while accounting for demographic, clinical, and micronutrient-related variables. A few important factors surfaced, which pointed to the intricate relationship between age, micronutrient status, and prostate health. Age showed a significant positive association with elevated PSA, with an adjusted OR of 1.05 (95% CI: 1.02–1.08; p = 0.002) per year of age, increasing the odds. In contrast, BMI was found to have no significant effect on the elevation of PSA (Adjusted OR = 1.02; p = 0.38), nor did co-existing diseases such as hypertension (Adjusted OR = 1.21; p = 0.44) or diabetes (Adjusted OR = 1.35; p = 0.27), thus proposing their minor role among the factors after adjustment in this cohort. Micronutrient deficiencies turned out to be strong independent predictors, still, however. Low serum zinc was the most strongly correlated factor, more than doubling the odds of elevated PSA (Adjusted OR = 2.48; 95% CI: 1.45–4.23; p = 0.001). Low selenium was associated with a significantly increased likelihood of high PSA (Adjusted OR = 2.12, 95% CI: 1.27–3.52; p = 0.004), followed by low vitamin D, which also remained an independent predictor (Adjusted OR = 1.87, 95% CI: 1.11–3.16; p = 0.018). There was no evidence of multicollinearity among zinc, selenium, and vitamin D (all variance inflation factor [VIF] <2) (Figure 1, Table 7).

**TABLE 7:** Logistic regression model performance and goodness-of-fit.

Outcome variable	Predictors included	C-statistic (AUC)	Hosmer–Lemeshow χ2 (df = 8)	p-value	Nagelkerke R2
PSA >4 ng/mL	Age, BMI, diabetes, family history, zinc, selenium, vitamin D	0.81	6.24	0.62	0.29
PSA >10 ng/mL	Age, BMI, diabetes, family history, zinc, selenium, vitamin D	0.84	5.71	0.68	0.33
Urinary retention (PVR >100 mL)	Age, BMI, diabetes, hypertension, zinc, selenium, and vitamin D	0.79	7.13	0.52	0.25

### 3.7 Association between micronutrient deficiency and severe urinary symptoms (IPSS > 20)

Separate associations between zinc, selenium, and vitamin D deficiencies and high PSA (>4 ng/mL) and severe urinary symptoms (IPSS ≥20) were observed. In the separate analyses, each micronutrient deficiency was associated with higher PSA, higher IPSS, lower Qmax, and higher PVR. This association was strongest for zinc, followed by selenium and, finally, vitamin D. To address potential heterogeneity within the composite group, individual micronutrient deficiencies were analyzed separately (Table 8).

**TABLE 8:** Association of individual micronutrient deficiencies with prostate outcomes.

Micronutrient deficiency	PSA >4 ng/mL (OR, 95% CI)	p-value	IPSS ≥20 (OR, 95% CI)	p-value
Zinc deficiency	2.52 (1.48–4.31)	0.001	2.24 (1.31–3.84)	0.003
Selenium deficiency	2.08 (1.22–3.61)	0.005	1.89 (1.07–3.27)	0.031
Vitamin D deficiency	1.83 (1.09–3.09)	0.020	1.76 (1.02–3.01)	0.042

### 3.8 Cumulative deficiency score analysis

There was a dose–response relationship between the number of micronutrient deficiencies and prostate health. As the score increased (2–3 deficiencies), the PSA, IPSS, and PVR increased, and Qmax decreased compared with persons without deficiencies. This finding supports the hypothesis of the cumulative negative impact of multiple micronutrient deficiencies. Data are presented in Table 9.

**TABLE 9:** Association between cumulative micronutrient deficiency score and prostate outcomes.

Deficiency score	PSA (ng/mL, mean ± SD)	IPSS (mean ± SD)	Qmax (mL/s)	PVR (mL)
0	2.7 ± 1.2	9.6 ± 4.7	16.7 ± 4.5	40 ± 16
1	3.4 ± 1.5	11.8 ± 5.1	15.2 ± 4.2	45 ± 17
2	4.3 ± 2.1	14.0 ± 5.6	13.0 ± 4.0	56 ± 19
3	5.7 ± 2.6	16.9 ± 6.0	10.8 ± 3.8	64 ± 20

A significant trend across deficiency scores was observed for all outcomes (p for trend <0.001).

**TABLE 10:** Subgroup Analysis by Age (<60 vs. ≥ 60 years).

Variable	<60 years	≥60 years	p-value
Zinc (μg/dL)	95.8 ± 17.2	89.5 ± 19.6	0.01
Selenium (μg/L)	114.2 ± 21.1	107.0 ± 22.8	0.002
Vitamin D (ng/mL)	30.2 ± 9.3	26.5 ± 9.3	<0.001
PSA (ng/mL)	2.9 ± 1.7	4.2 ± 2.3	<0.001
IPSS	10.8 ± 5.6	13.5 ± 6.7	<0.001
Qmax (mL/s)	16.0 ± 4.5	13.5 ± 5.1	<0.001
PVR (mL)	42.0 ± 18.7	54.0 ± 22.1	<0.001

### 3.9 Multivariable regression for severe urinary symptoms (IPSS ≥20)

Multivariable logistic regression analysis for severe urinary symptoms (IPSS ≥20), which were described by an International Prostate Symptom Score (IPSS) of 20 or more. Age was by far the most important predictor, as the odds of developing severe symptoms increased by 3% for each additional year (Adjusted OR = 1.03; 95% CI: 1.01–1.06; p = 0.012). The analysis for body mass index, on the other hand, concluded that his was no significant impact on the severity of urinary symptoms (Adjusted OR = 1.01; p = 0.70), thus indicating that BMI was not a major determinant in this population group.

The deficiencies in micronutrients once more pointed out the undeniable linkages to unfavorable results. Zinc deficiency in the body was the strongest predictor by more than 100% increase in the odds of severe urinary symptoms (Adjusted OR = 2.20; 95% CI: 1.28–3.78; p = 0.004). The condition of Selenium deficiency also had a significant impact on the odds of severe urinary symptoms (Adjusted OR = 1.85; p = 0.033), implying that sufficient Selenium status may boost urinary function. Further, low levels of Vitamin D were also identified as an independent predictor (Adjusted OR = 1.73; 95% CI: 1.01–2.97; p = 0.045), underscoring its potential role in minimizing symptom severity.

**FIGURE 2:**
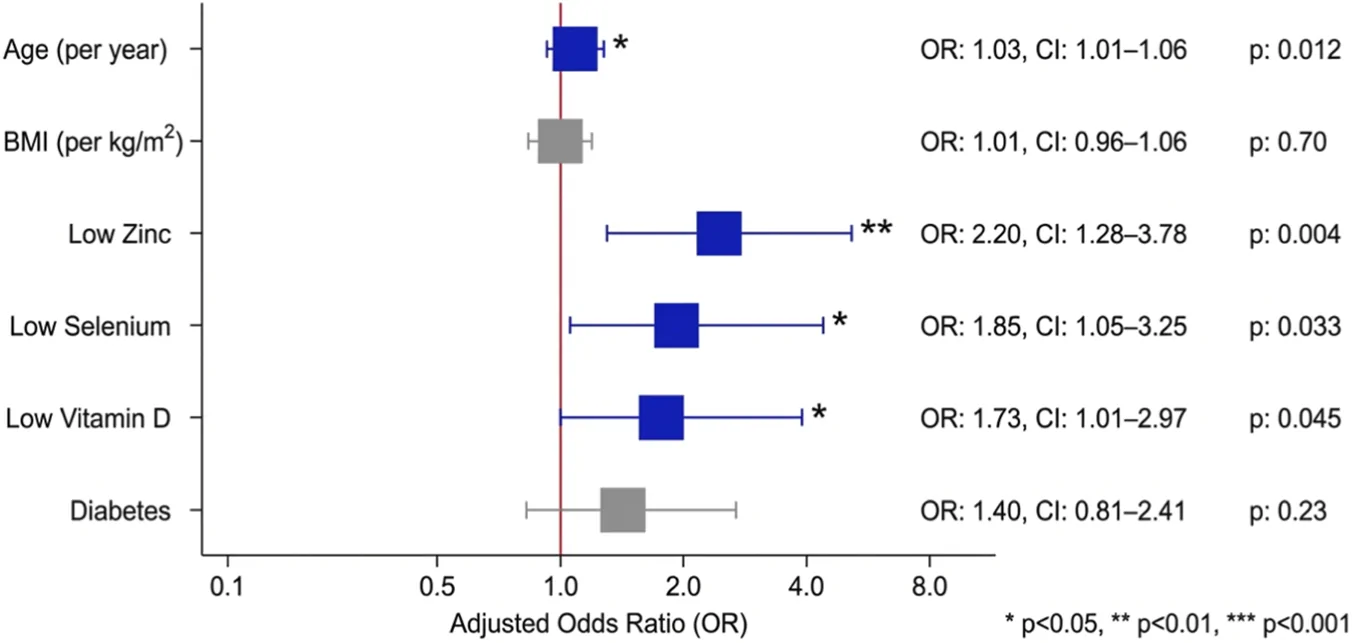
Multivariable regression for severe urinary symptoms (IPSS ≥20).

Diabetes was associated with a non-significant trend towards raising the odds of developing severe symptoms (Adjusted OR = 1.40; p = 0.23), but this relationship was not robust enough to be considered statistically significant after adjustment (Figure 2).

### 3.10 Subgroup analysis by age group

The analysis of subgroups based on age, that is, the comparison between the participants younger than 60 years and the people aged 60 years and above, showed the differences related to age in terms of micronutrient status, prostate biomarkers, and urinary tract function to be very significant. The serum micronutrient levels of the younger participants (<60 years) were consistently higher than the levels of all measured nutrients. The younger group (95.8 ± 17.2 μg/dL) had significantly higher mean zinc levels than the older group (89.5 ± 19.6 μg/dL; p = 0.01). A similar trend was observed for selenium concentrations, which were lower among participants below 60 (114.2 ± 21.1 μg/L) than among those aged 60 and above (107.0 ± 22.8 μg/L; p = 0.002). Vitamin D also levels when compared to younger adults (30.2 ± 9.3 ng/mL; p < 0.001) significantly decreased with age, and older adults could only show (26.5 ± 9.3 ng/mL) as their levels (Table 10).

**FIGURE 3:**
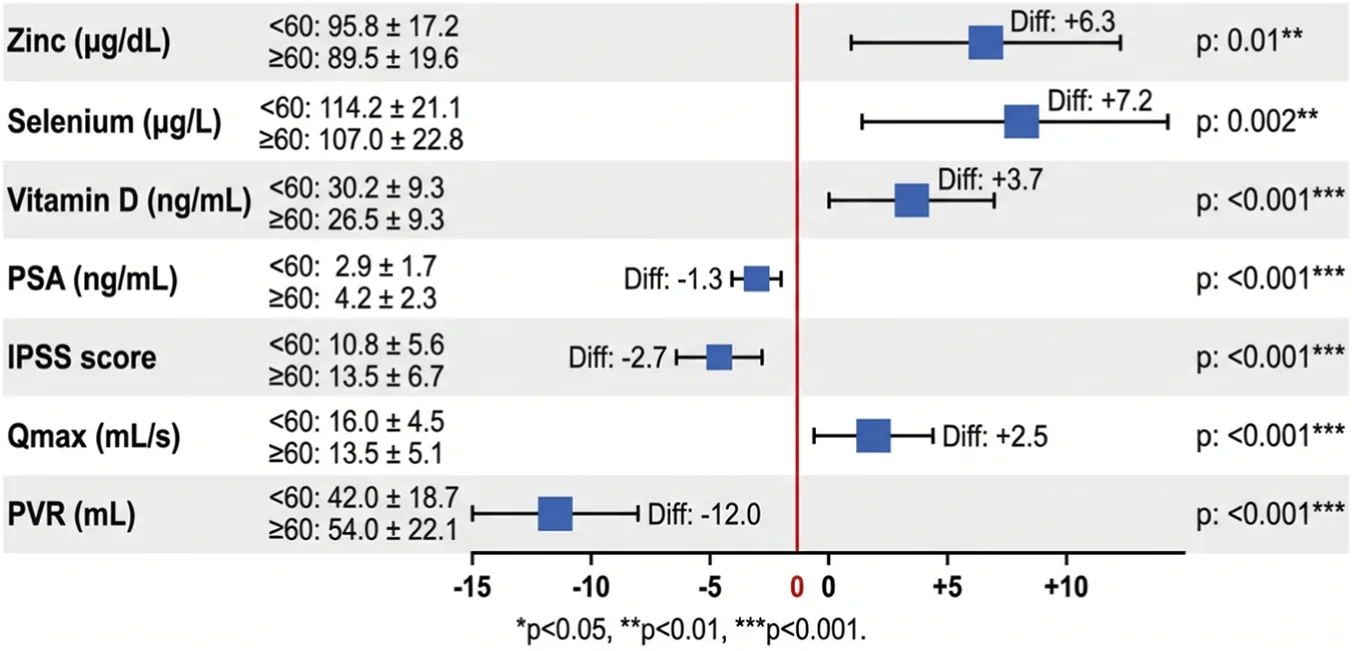
Subgroup Analysis by Age Group (<60 vs. ≥ 60 years).

The health indicators of the prostate also showed a similar trend of age-related changes. Among older adults, PSA level was (4.2 ± 2.3 ng/mL) and was significantly higher as compared to the younger group (2.9 ± 1.7 ng/mL; p < 0.001), hence a suggestion of a larger prostate and/or existing pathology among older individuals. The results of the subgroup analysis by age group are summarized in Table 8 and shown in Figure 3.

**FIGURE 4:**
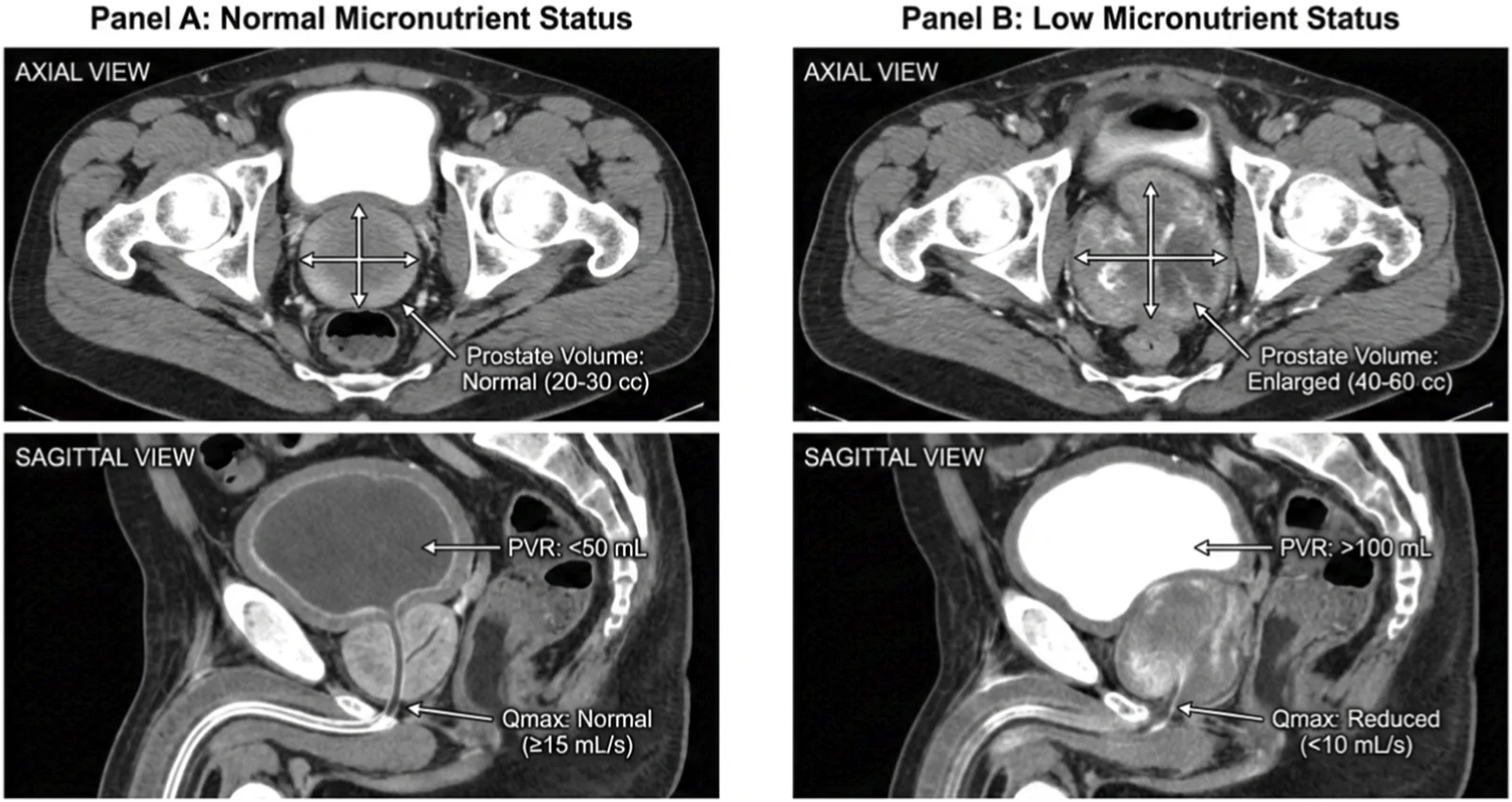
Contrast-Enhanced CT Scans of the Prostate and Lower Urinary Tract. **(A)** shows normal anatomy and function. **(B)** illustrates signs of prostatic enlargement and partial obstruction associated with low micronutrient status. PVR, Post-Void Residual Volume; Q_max_, Maximum Urinary Flow Rate. The CT scans are displayed only for illustrative purposes to show typical anatomical structures and were not used in calculations.

Urinary function also deteriorated with age. Elderly subjects displayed a greater intensity of urinary symptoms which is clearly seen through the reported IPSS scores (13.5 ± 6.7 vs. 10.8 ± 5.6; p < 0.001) as well as through the measures of peak urinary flow rates where they had a lower value (Qmax: 13.5 ± 5.1 vs. 16.0 ± 4.5 mL/s; p < 0.001) and post-void bladder volumes that were larger (54.0 ± 22.1 vs. 42.0 ± 18.7 mL; p < 0.001) (Figure 4).

## 4 Discussion

In the current study, we report associations of micronutrient status with prostate-related parameters. In particular, low serum levels of selenium and vitamin D were associated with increased odds of severe lower urinary tract symptoms (LUTS) and prostate-specific antigen (PSA) after controlling for potential confounders. Descriptive statistics showed that the baseline characteristics of participants with low and normal micronutrient status also differed, and low levels of zinc, selenium, and vitamin D were associated with age, metabolic conditions, and less healthy lifestyles. Men with low micronutrient status were older, consistent with prior studies showing that older age is associated with lower levels of antioxidant micronutrients. This finding is in line with observations by Pietrzak et al. (2024) and Jin et al. (2025), where reduced antioxidant micronutrient status has been associated with increased prostate cancer risk, particularly among middle-aged and older men.

These trends may be due to decreased dietary intake, absorption, and vitamin D production with age. Further, the low micronutrient group had a slightly higher BMI, consistent with Thederan et al. (2021), who found lower levels of circulating vitamin D and selenium in obese patients with prostate cancer. This may suggest a link between metabolic dysfunction, obesity-associated inflammatory processes, and micronutrient status. Serum micronutrient profiling demonstrated significantly lower zinc, selenium, and vitamin D levels in the low-micronutrient group than in the normal group, with all values below the minimum reference levels. Lower zinc levels detected in this group are consistent with previous reports of an association between low zinc and dysfunction of the prostate gland.

Karunasinghe (2022) reported that the prostate gland is highly dependent on zinc for normal metabolic and antioxidant activity. At the same time, a meta-analysis by Nejad et al. (2024) found that low levels of zinc were associated with prostate cancer. Likewise, Dhillon et al. (2022) reported lower zinc levels in the serum of patients with prostate cancer than in controls. These findings are consistent with the present findings. Similar results were also observed for selenium, with lower concentrations observed in the low micronutrient group. This observation is in line with previous studies showing that lower selenium status is linked to compromised antioxidant status in prostate tissues. Karunasinghe et al. (2019) observed associations between selenium status and prostate health biomarkers in patients with low selenium status who were supplemented with selenium, while Dhillon et al. (2022) also reported lower selenium levels in prostate cancer cases than in controls. While several mechanisms have been proposed to explain the link between micronutrient status and prostate health, including oxidative stress and inflammation, the current study did not report molecular or biomarker data; therefore, the findings remain correlational. While common pathways linking micronutrient status and prostate health include oxidative stress and inflammation, this study did not directly measure molecular or biomarker targets. As such, these inferences should be interpreted as hypothesis-generating statements.

The greatest difference between the groups was in vitamin D concentrations, which were significantly lower in the low-micronutrient group than in the sufficient group. This is in line with previous reports by Brown et al. (2022), which found links between vitamin D status and benign prostatic hyperplasia, chronic prostatitis, lower urinary tract symptoms, and aggressiveness of prostate cancers. This result is in line with a general pattern of association between low vitamin D levels and poor prostate outcomes. Among PSA, the group with low micronutrient status had significantly higher PSA levels than the group with normal micronutrient status. The number of participants with abnormal PSA levels (>4 ng/mL and >10 ng/mL) was also greater in this group.

These results are in line with those of Olooto et al. (2021), who observed increased PSA levels and low micronutrient status in men with prostate carcinoma. David and Leslie (2024) also reported that higher PSA levels are typically found in conditions of prostate inflammation, benign prostatic hyperplasia, and prostate cancer, which may be associated with decreased antioxidant micronutrient status. Zhu et al. (2025) also reported that exposure to environmental and toxic factors is associated with reduced levels of protective micronutrients such as zinc and selenium, which may be linked to biochemical changes in PSA levels. In the current study, the reported association between low micronutrient status and higher PSA categories suggests a consistent relationship between low antioxidant micronutrient status and prostate-specific biomarker levels.

Patterns of association with urinary tract parameters: Those in the low micronutrient group had higher International Prostate Symptom Score (IPSS) and. Those in the low micronutrient group had higher International Prostate Symptom Score (IPSS), lower peak urinary flow rate (Qmax), and higher post-void residual (PVR) volumes than those in the normal micronutrient group. These results suggest an association between low micronutrient concentrations and worse lower urinary tract symptoms. Da Leo et al. (2022) also found associations between higher PSA levels, increased prostate volume, and decreased urinary flow rate in benign prostatic hyperplasia, which were consistent with those found in this study. Low levels of zinc, selenium, and vitamin D have also been found in other studies to be associated with inflammatory changes that may occur in parallel with stromal hyperplasia and urinary obstruction.

Additionally, Sakalis et al. (2021) found associations between prostate volume, PSA, urinary flow rate and residual urine volume, which are consistent with findings from our data, where lower Qmax and increased PVR accompanied higher PSA levels in the low micronutrient group. Taken together, the current results suggest that there are consistent associations between low serum concentrations of zinc, selenium, and vitamin D and poor prostate-related outcomes, such as greater PSA level, higher symptom domains, and decreased urinary flow parameters. These findings are consistent with earlier reports of the antioxidant and anti-inflammatory actions of these micronutrients. Jin et al. (2025) found that greater antioxidant consumption is linked to decreased risk of high-grade prostate cancer, consistent with the idea of an oxidative balance in prostate health.

Likewise, Messina et al. (2022) reported associations between the consumption of dietary bioactive compounds with antioxidant properties and better prostate health. Zinc was the most significant micronutrient associated with PSA elevation in multivariable regression, with low zinc status associated with increased odds of PSA elevation. This result is in line with Radhi et al. (2023), who found inverse associations between serum zinc and PSA in men with benign prostatic hyperplasia. Similarly, low selenium status was associated with elevated PSA levels, which is in line with dietary studies that have reported inverse associations between dietary intake of selenium and prostate cancer. Vitamin D also showed significant, but weaker, associations with prostate health. This is in line with reports from Pan et al. (2025), who described associations between adequate vitamin D status and prostate health, and from Panda et al. (2024), who reported the antiproliferative and anti-inflammatory properties of vitamin D in prostate tissue.

### 4.1 Strengths

This piece of work has several strengths that make it a good scientific piece. First, the sample size was large (400 participants), which provided adequate statistical power to identify significant relationships between micronutrient status and prostate health outcomes. Second, the cohort study took an overall measurement of various serum micronutrients, zinc, selenium, and vitamin D, and subjective (IPSS) and objective (Qmax, PVR) urinary tract functioning, enabling a complex analysis of prostate health. Third, the study reduced the chances of confounding variables that might have biased the correlation between the levels of micronutrients and prostate outcomes by eliminating those with a prior history of prostate cancer, acute or chronic infections, and recent supplementation. Additionally, multivariable regression analyses were used to adjust for the main demographic and clinical covariates (age, BMI, diabetes, hypertension, and family history), thereby enhancing the identification of independent predictors. Lastly, the age-specific analysis created layers of age-related trends in interpreting the results and assessing the significance of micronutrient adequacy among older people.

### 4.2 Limitations

Despite these strengths, there are a few limitations of the study that are to be taken into consideration. Retrospective design, by its nature, restricts causal inference because it is impossible to determine a causal relationship between micronutrient status, on the one hand, and prostate outcomes, on the other hand, at the temporal level. The use of medical records can also introduce bias, especially when lab or clinical test results were not properly recorded. The second limitation is that residual confounding from lifestyle factors (diet, physical activity, and alcohol intake) that were not always documented may affect micronutrient levels or prostate conditions. Moreover, the study population was sampled from a single tertiary urology center, which could limit the generalizability of the findings to the wider population. Lastly, the exclusion of patients receiving micronutrient supplementation, although necessary to limit confounding, can introduce selection bias and underestimate the potential efficacy of supplementation in practice. This study is subject to several limitations, such as the retrospective design precluding inference of causality, and prostate volume, medication use, inflammatory biomarkers, renal function and other lifestyle factors were not available, raising a possibility of residual confounding. Then, the data are from a single centre, which may limit generalizability and micronutrient status was measured at a single point in time and may not reflect long-term status. No formal multiplicity correction was applied in the original analysis because the study was exploratory in nature and focused on hypothesis generation rather than confirmatory testing. Prostate disease severity may contribute to lifestyle modifications, altered dietary intake, reduced physical activity, or decreased sunlight exposure, subsequently affecting micronutrient status.

### 4.3 Future recommendations

Future studies ought to explore the weaknesses of the present study by using prospective cohort or interventional studies to elucidate the causation between the status of micronutrients and prostate-related health. The trials that are randomised and controlled and evaluate the impact of zinc, selenium, and vitamin D supplementation on the PSA levels and the functioning of the urinary tract would be more convincing when it comes to therapeutic advice. Further, the intake of diet evaluation, exercise measurements, and more general sociodemographic variables would go a long way in managing any residual confounding, as well as providing a more globalized perspective on the effects of micronutrients. By extending the research to institutions in different populations in Chines and beyond, the external validity of the results would be enhanced and inform the efforts of health authorities. Lastly, the longitudinal observation of prostate volume and clinical progression and the levels of micronutrients can contribute to the explanation of the biological mechanisms of the observed associations.

## 5 Conclusion

This study provides compelling evidence that deficiencies in serum vitamin D, selenium, and zinc are significantly associated with elevated PSA levels and increased urinary tract dysfunction among men aged 45 years and older. Of these micronutrients, zinc deficiency was the strongest independent predictor of increased PSA as well as severe urinary symptoms, and thus plays a crucial role in prostate health. The results also highlight the impact of age on micronutrient status and prostate outcomes, underscoring the significance of early monitoring and nutritional intervention in the aging population. The results suggest strong associations between micronutrient deficiencies and prostate health markers, but cannot establish causality given the retrospective nature of this study. Additional prospective and intervention studies are needed.

## Data Availability

The raw data supporting the conclusions of this article will be made available by the authors, without undue reservation.
